# High-Dose Therapy and Autologous Hematopoietic Progenitor Cells Transplantation for Recurrent or Refractory Hodgkin's Lymphoma: Analysis of King Hussein Cancer Center Results and Prognostic Variables

**DOI:** 10.5402/2012/249124

**Published:** 2012-02-14

**Authors:** Fawzi Abdel-Rahman, Ayad Hussein, Mohammad Aljamily, Abdulhadi Al-Zaben, Nilly Hussein, Ala'a Addasi

**Affiliations:** ^1^Bone Marrow and Stem Cell Transplantation Program, King Hussein Cancer Center, P.O. Box 1269 Al-Jubeiha, Amman 11941, Jordan; ^2^Department of Internal Medicine, King Hussein Cancer Center, Amman 11941, Jordan

## Abstract

*Purpose*. to evaluate the outcome of patients with Hodgkin's lymphoma who underwent autologous transplantation at KHCC bone marrow transplant program. *Patients and Methods*. Over 6 years, 63 patients with relapsed or refractory Hodgkin's lymphoma underwent high dose chemotherapy followed by autologous transplant. There were 25.4% patients in complete remission (CR), 71.4% with chemotherapy responsive disease at the time of transplant. Prior to conditioning regimen, 56% received two chemotherapy lines, and, 44% received more than two lines. *Results*. The main outcomes of the study are the rate of complete remission at day 100, overall survival (OS), relapse-free survival (RFS), The impact of the following variables on OS and RFS: (a) disease status at the time of transplant, (b) number of chemotherapy lines prior to conditioning, (c) age group, (d) time of relapse < or >12 months were investigated. The CR at day 100 was 57%. The median overall survival for the whole group was 40.6 months; the median RFS was 20 months. The only factor which significantly impacts the study outcomes was the number of chemotherapy lines prior to conditioning on OS in favor of patients received two lines. *Conclusion*. In our study only the number of chemotherapy lines received before conditioning had statistically significant impact on OS.

## 1. Introduction

Among patients with early-stage Hodgkin's lymphoma (HL) the rate of relapse ranges from 10 to 15% [1] and in patients with advanced stage HL the relapse rate ranges from 30 to 40% [2–6]. In addition, approximately 10–15% of patients experience progression of disease after an initial partial response.

High-dose chemotherapy (HDCT) followed by hematopoietic stem cell transplantation can achieve sustained remission in patients with advanced refractory or recurrent HL [7–14]. Several factors can predict survival and RFS after high-dose chemotherapy and autologous transplant. One of these factors is the number of chemotherapy lines patients received prior to transplant [15–19]. Another factor which was shown in previous studies to predict the risk of relapse was the disease status at the time of transplant, with superior outcomes in patients who were in complete remission (CR), or minimal residual disease in comparison to those with bulky disease [14, 17, 20], and the last factor which was shown to influence the outcome is the time to initial relapse (TTR) >12 months versus <12 months which is measured from the date of diagnosis to that of proven relapse [21]. At the King Hussein Cancer Center (KHCC) bone marrow and stem cell transplantation program, Amman, Jordan, we performed stem cell transplantation for 63 patients with HL between the years 2003 and 2008. We report herein the outcome of these patients. Since our program is combined for adult and pediatric patients, our study includes, as one of its objectives, the comparison between the two age groups in treatment outcome. To our knowledge there is no data available on the impact of age on transplantation outcome of HL. (Adult patients at our center are defined as 18 years or older.) 

## 2. Patients and Method

The outcome of sixty-three patients who underwent HSCT at KHCC between January 2003 and December 2008 was studied through retrospective chart review after obtaining IRB approval.

The diagnosis was confirmed for all patients at KHCC by pathology review, and they all met the standard criteria to be eligible for autologous transplant. We included all patients who underwent transplant if all data required for the study was available in their files.

We reviewed 70 files for patients with relapsed/refractory HL who underwent transplant at KHCC during the study period. Data about disease status at the time of transplant, number of chemotherapy lines received before transplant, evaluation at day 100, vital status, and disease status at last followup was available for sixty-three patients (90%), so this group composed our study population.

### 2.1. Treatment

Prior to the conditioning regimen 56% of the patients received only two lines of chemotherapy, and 44% received more than two lines. Chemotherapy and GCSF mobilization followed by stem cell collection was used in 89% of patients, while GCSF alone followed by stem cell collection was used in 11% of cases. The source of stem cells was peripheral blood in all cases.

Sixty (95%) of 63 patients received BEAM (BCNU, Etoposide, Ara-C, and Melphalan) as conditioning. One patient received CBV (Cytoxan, BCNU, VP16), and two patients received TEAM (Thiotepa, Etoposide, Ara-C, and Melphalan). The median CD34 dose delivered was 5.6 × 10^6^/kg (1.51–12.1). All patients received routine prophylaxis with acyclovir, fluconazole, and trimethoprim/sulfamethoxazole.

Patients had followup CT scan at day 30, and CT scan or PET-CT scan at day 100. RECIST criteria were used to assess response by CT scan. The majority of patients had only CT scan for followup.

### 2.2. End Points

The primary end points were

- the rate of complete remission at day 100,
- overall survival (OS),
- relapse-free survival (RFS),
- overall survival and RFS according to disease status at the time of transplant, number of chemotherapy lines, age group, and TTR.

The secondary end points were

- day 100 nonrelapse mortality (NRM),
- incidence of grade 3-4 mucositis.

### 2.3. Statistical Analysis

The rate of complete remission at day 100, as well as the overall survival, and relapse-free survivals were the primary outcomes of this study. Furthermore, day 100 nonrelapse mortality (NRM) and incidence of grade 3, 4 mucositis are secondary outcomes to be investigated in this study. Outcome comparison between groups categorized by disease status at the end of treatment, number of lines of chemotherapy, time to relapse, and age groups was carried out. Kaplan-Meier method was used to present survival curves. Comparison between groups in survival was carried out using Log Rank test.

## 3. Results

### 3.1. Patients' Characteristics

The characteristics of the 63 patients at the time of autologous stem cell transplant are listed in Table 1.

Only 16 (25.4%) of the 63 patients were in complete remissions (CRs) at the time of transplantation; the majority of patients in this series had responsive disease, 45 patients (71.4%), and 2 patients (3%) had stable disease. All patients had successful engraftment, with a median time of 10 days for white blood cells and 12 days for platelets engraftment.

#### 3.1.1. Overall Survival

**Figure 1:**
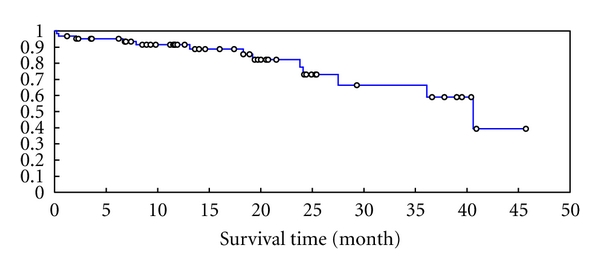
Overall survival of 63 patients received autologous transplantation for HL.

All 63 patients were followed with a median follow-up time of 34.89 months. The median overall survival was 40.6 months, with projected 3-year overall survival of 64.6% as shown in Figure 1 and Table 2.

**Figure 2:**
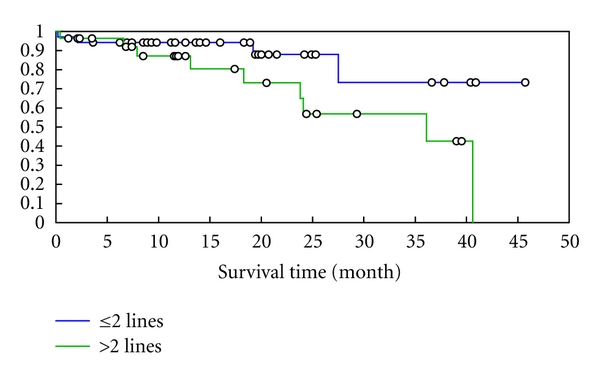
Overall survival of 63 patients with HL according to number of lines of chemotherapy received before transplantation.

The overall survival of patients who received two lines before transplant was significantly better than that of those who received more than two lines, 73% versus 43% (*P* = 0.049) Figure 2 and Table 3. However, there was no statistically significant difference between those who were in complete remission versus those with responsive disease. Also, there was no statistical difference in the survival of children or adults who received autologous transplant and no difference in survival according to TTR >12 months or <12 months.

#### 3.1.2. Relapse-Free Survival

**Figure 3:**
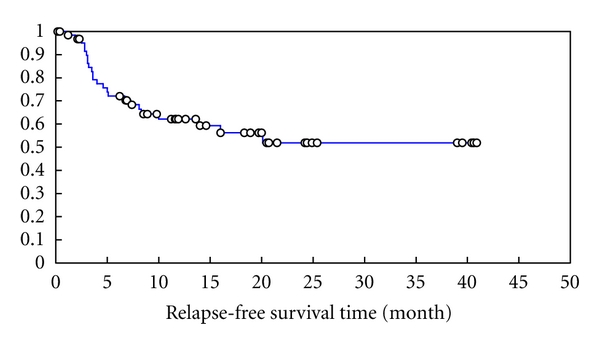
Relapse free-survival of 63 patients received autologous transplantation for HL.

**Figure 4:**
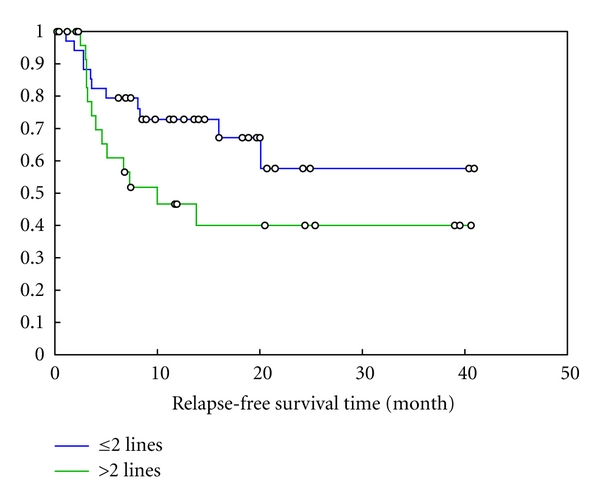
Relapse-free survival of 63 patients with HL according to number of lines of chemotherapy received before transplantation.

The median RFS for the whole group was 20 months. The projected 3-year RFS is 42.3% Figure 3 and Table 4. Exploring the impact of disease status at transplant, number of lines of chemotherapy age group, and TTR on relapse-free survival, it was found that none of these factors significantly affected the time to relapse. It is worth mentioning here that there was a trend for improvement in RFS for patients who received two lines in comparison to those who received more than two (*P*  value = 0.095) as seen in Figure 4 and Table 5.

### 3.2. Day 100 Mortality and Incidence of Grade 3-4 Mucositis

Only three patients died in the first 100 days (4.8%), after transplant. The cause of death was sepsis in all of the three cases. This is comparable to the acceptable international figures. As for grade 3-4 mucositis, its occurrence reached 50%.

## 4. Discussion

High-dose chemotherapy with autologous stem cell transplantation is a well-established potentially curative therapy for relapsed/refractory Hodgkin's lymphoma.

At KHCC sixty-three patients underwent high-dose chemotherapy with autologous transplant from 2003 to 2008. At the time of transplantation 25% of patients were in CR, and at day 100 the number rose to 57%. Prior to the conditioning regimen, 55.6% received two lines of chemotherapy and 44.4% received more than two lines.

The median survival for the whole group was 40.6 months, with projected 3-year overall survival of 64.6%, and the median RFS was 20 months, with projected 3-year RFS of 42.3%.

These results were close to the 5-year OS and RFS published by Engelhardt et al. [21].

All patients engrafted on time. The incidence of grade 3-4 mucositis was 50%, and the day 100 mortality was 4.8%.

The correlation between the numbers of chemotherapy lines received prior to conditioning and survival was statistically significant. This is consistent with some previously published studies [15–19], although this was not shown in the study published by Engelhardt et al. [21]. In our study there was a trend towards improvement in RFS in patients who received two lines of chemotherapy in comparison to those who received more than two lines, but this was not statistically significant, probably due to the small sample size.

The disease status at the time of transplant (CR versus RD) did not affect the OS or RFS, and this is consistent with Engelhardt et al. study [21], but contradicting other studies [14, 17, 20]. We believe that the main reason for this is the small sample size in our study, as there was a trend towards improvement in patients who were in CR over those who had RD, with *P* values of 0.10 and 0.15 for RFS and OS, respectively.

There was no difference in outcome between the two age groups in term of OS and RFS, with the caveat of the small number of pediatric patients. There was no difference in OS and RFS according to TTR.

## 5. Conclusion

We can therefore conclude from our study that the number of chemotherapy lines received prior to the conditioning regimen is the most important predictor of survival.

Our patients who proceeded to autologous transplantation had to have chemosensitive disease; otherwise changing the chemotherapy line was recommended in case progression or no response after two cycles of therapy. This supports the hypothesis that the tumor biology is the most important predictor of OS, with tumors that were sensitive to the first line of salvage chemotherapy having a better outcome than those that were not, even if the latter responded to further salvage chemotherapy.

Relapse after autologous transplant for HL remains a significant problem with more than 50% of patients relapsing as projected from our study, so it is important to focus on treatment strategies after relapse.

There is a potential durable response with reduced-intensity allogenic transplant for HL patients relapsed after autologous transplant as shown by the study of Peggs et al. [22].

Novel agents like Brentuximab Vedotin are promising with phase II studies showing response rate as high as 75% in relapse/refractory HL. It is currently under investigation in phase III trial for patients with HL at high risk for residual disease following autologous stem cell transplantation [23].

## Figures and Tables

**Table 1:** Characteristics of 63 patients with HL who underwent autologous stem cell transplantation.

		Count (%)
Gender	Male	38 (60.3%)
	Female	25 (39.7%)
Age	Adult	54 (85.7%)
	Pediatric	9 (14.3%)
Number of previous Chemotherapy lines received	2 lines	35 (55.6%)
	>2 lines	28 (44.4%)
Disease status at transplant	Complete remission	16 (25.4%)
	Responsive disease	45 (71.4%)
	Stable disease	2 (3.2%)
Time to relapse (TTR)	≤12 months	30 (48%)
	>12 months	33 (52%)
Mobilization regimen	Chemotherapy + GCSF	57 (90.5%)
	GCSF only	6 (9.5%)
Stem cell source	Peripheral	63 (100%)
Stem cell dose	Median (range)	5.6 × 10^6^/kg
		(1.51−12.1 × 10^6^/kg)
Conditioning regimen	BEAM*	60 (95.2%)
	TEAM**	2 (3.2%)
	CBV***	1 (1.6%)

*BEAM: BCNU, etoposide, Ara-C, melphalan.

**TEAM: thiotepa, etoposide, Ara-C, melphalan.

***CBV: cyclophosphamide, BCNU, VP16.

**Table 2:** 

Summary statistics	
Total observed	63
Total failed	13
Total censored	50

Mean survival time	
Mean survival time (survival < 40.9)	32.550
Standard deviation	2.070
Lower bound (95%)	28.492
Upper bound (95%)	36.608

Quantiles estimation	
Quantile	50%
Estimate	40.600
Lower bound (95%)	27.500
Upper bound (95%)	

**Table 3:** 

Summary statistics (≤2 line)	
Total observed	35
Total failed	4
Total censored	31

Quantiles estimation (≤2 line)	
Quantile	50%
Estimate	
Lower bound (95%)	
Upper bound (95%)	

Quantiles estimation (>2 lines)	
Total observed	28
Total failed	9
Total censored	19
Quantile	50%
Estimate	36.100
Lower bound (95%)	23.800
Upper bound (95%)	40.600

Statistic	Log-rank
*P* value	0.050

**Table 4:** 

Summary statistics	
Total observed	63
Total failed	24
Total censored	39

Quantiles estimation	
Quantile	50%
Estimate	
Lower bound (95%)	NA
Upper bound (95%)	NA

**Table 5:** 

Summary statistics (≤2 line)	
Total observed	35
Total failed	11
Total censored	24

Quantiles estimation (≤2 line)	
Quantile	50%
Estimate	NA
Lower bound (95%)	
Upper bound (95%)	

Summary statistics (>2 lines)	
Total observed	28
Total failed	13
Total censored	15

Quantiles estimation (>2 lines)	
Quantile	50%
Estimate	10.000
Lower bound (95%)	4.600
Upper bound (95%)	

Test of equality of the survival distribution functions (DF = 1)	
Statistic	Log-rank
*P* value	0.094
